# HIV is associated with endothelial activation despite ART, in a sub-Saharan African setting

**DOI:** 10.1212/NXI.0000000000000531

**Published:** 2018-12-21

**Authors:** Joseph Kamtchum-Tatuene, Henry Mwandumba, Zaid Al-Bayati, Janet Flatley, Michael Griffiths, Tom Solomon, Laura Benjamin

**Affiliations:** From the Institute of Infection and Global Health (J.K.-T., Z.A.-B., J.F., M.G., T.S., L.B.), University of Liverpool; Malawi-Liverpool-Wellcome Trust Clinical Research Programme (J.K.-T., H.M.), University of Malawi College of Medicine, Blantyre; Department of Clinical Sciences (H.M.), Liverpool School of Tropical Medicine, United Kingdom.

## Abstract

**Objective:**

To study the relationship between endothelial dysfunction, HIV infection, and stroke in Malawians.

**Methods:**

Using a cross-sectional design, we measured plasma levels of intercellular adhesion molecule-1 (ICAM-1), plasminogen activator inhibitor-1 (PAI-1), vascular endothelial growth factor (VEGF), and soluble thrombomodulin (sTM) in stroke patients and controls, stratified by HIV status. These biomarkers were measured using ELISA. After dichotomization, each biomarker was used as the dependent variable in a multivariable logistic regression model. Primary independent variables included HIV and stroke status. Adjustment variables were age, sex, hypertension, diabetes mellitus, tobacco and alcohol consumption, personal/family history of stroke, antiretroviral therapy status, and hypercholesterolemia.

**Results:**

Sixty-one stroke cases (19 HIV+) and 168 controls (32 HIV+) were enrolled. The median age was 55 years (38.5–65.0) for controls and 52 years (38.0–73.0) for cases (*p* = 0.38). The median CD4^+^ T-cell count was 260.1 cells/mm^3^ (156.3–363.9) and 452 cells/mm^3^ (378.1–527.4) in HIV-infected cases and controls, respectively. HIV infection was independently associated with high levels of ICAM-1 (OR = 3.6, 95% CI: 1.3–10.6, *p* = 0.018) in controls but not in stroke cases even after excluding patients with a viral load >1,000 RNA copies/mL (OR = 4.1, 95% CI: 1.3–13.1, *p* = 0.017). There was no association between the clinical profiles of HIV-positive controls or HIV-positive stroke and high levels of PAI-1, VEGF, and sTM.

**Conclusions:**

HIV infection is associated with endothelial activation despite antiretroviral treatment. Our findings underscore the need for larger clinical cohorts to better understand the contribution of this perturbation of the endothelial function to the increasing burden of cardiovascular diseases in sub-Saharan Africa.

The vascular endothelium is a critical regulator of cell adhesion, inflammation, coagulation, and vascular remodeling. Perturbation of one or more of these functions, also known as endothelial dysfunction, is induced by classical cardiovascular risk factors. Endothelial dysfunction is a major component of local and systemic inflammation. At the early stage of endothelial activation, there is increased expression of cell adhesion molecules (e.g., intercellular adhesion molecule-1 [ICAM-1]) on the surface of endothelial cells to facilitate leukocyte transendothelial migration. The occurrence of endothelial damage leads to the secretion of other biomarkers, notably plasminogen activator inhibitor-1 (PAI-1) and soluble thrombomodulin (sTM) responsible for the perturbation of the coagulation process and vascular endothelial growth factor (VEGF) responsible for angiogenesis.^1^ The interaction of these biomarkers with other proinflammatory cytokines, as well as white blood cells and smooth muscle cells, leads to vessel wall remodeling and ultimately to various types of vasculopathy.

Several studies conducted on HIV populations from Western countries have reported an association between high levels of biomarkers of endothelial dysfunction and death and/or cardiovascular endpoints.^2^ However, it is unknown whether the same association is observed in a sub-Saharan African setting, where antiretroviral therapy (ART) has become more widespread. This knowledge gap provides the rationale for this study conducted to evaluate the relationship between endothelial dysfunction, HIV infection, and stroke in Malawi.

## Methods

### Standard protocol approvals, registrations, and patient consents

This work was conducted as part of the study of Biomarkers of Stroke and Arterial Diseases in Malawian Adults, which was approved by the Research Ethics Committees of the Liverpool School of Tropical Medicine and the University of Malawi College of Medicine. The study used plasma samples available in the biobank of the Malawi START study, a case-control study designed to identify risk factors of stroke in Malawi, where the prevalence of HIV infection is 10.3%.^3^ All patients enrolled in the Malawi START study provided a written informed consent that allowed their blood specimens to be reused for subsequent ethically cleared studies within a 5-year period from the time of collection.

### Patient selection

As previously described,^3^ stroke cases enrolled in the original Malawi START study were adult residents of Blantyre presenting at the Queen Elizabeth Central Hospital within 7 days of symptom onset, meeting the WHO's clinical definition of stroke, and having a brain MRI confirming the diagnosis. Two community controls were selected for each case with the goal of obtaining an age/sex frequency-matched random sample of the population, with a geographical distribution in proportion to the population density. The subsequent patient selection (or more accurately “plasma specimen selection”) for the current study is described in figure.

**Figure:**
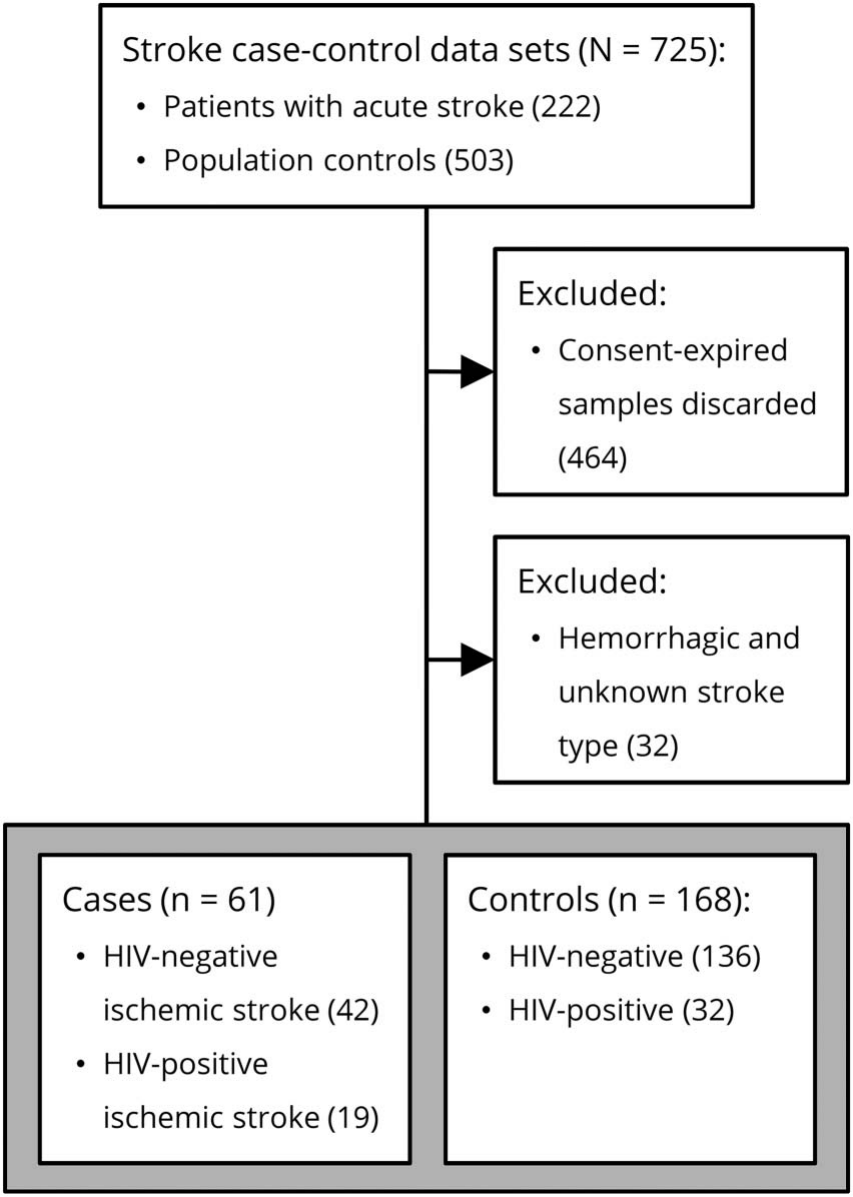
Flow diagram of case and control selection

Demographic and clinical data of cases and controls included age, sex, hypertension, diabetes status, tobacco and alcohol consumption status, personal/family history of stroke, CD4^+^ T-cell count, HIV viral load, hypercholesterolemia, ART status, recent infection, and National Institute of Health Stroke Score for stroke cases only.^3^ The stroke type and etiology were defined using MRI brain imaging and the trial of Org 10172 in acute stroke treatment criteria, respectively.^3^

### Selection of biomarkers of endothelial dysfunction

The biomarkers measured in this study were selected to assess the different stages of endothelial dysfunction as described in the introduction. The major cellular source of ICAM-1, PAI-1, and thrombomodulin is activated endothelial cells.^4^ VEGF, an essential growth factor for endothelial cells, is produced by macrophages, platelets, and keratinocytes. There is no validated normal range for the plasma concentration of biomarkers of endothelial dysfunction. Higher concentrations correlate well with the magnitude of local or systemic inflammation and have been consistently associated with an increased cardiovascular risk. Because HIV-infected individuals have persistent systemic inflammation,^5^ we hypothesized that they would have higher levels of biomarkers of endothelial dysfunction, explaining their greater risk of stroke.

### Measurement of biomarkers of endothelial dysfunction

The plasma concentrations of ICAM-1, PAI-1, VEGF, and sTM were measured using ELISA kits (Abcam, Cambridge, United Kingdom). SimpleStep ELISA kits were used for ICAM-1, PAI-1, and sTM, whereas the standard kit was used for VEGF. All samples were run in duplicates. Samples with an individual coefficient of variability (CV) greater than 25% were reanalyzed.

All the ELISA tests were performed according to the manufacturer’s instructions with the exception of the washing steps that were performed with an automated washer (Wellwash; ThermoFisher Scientific, Waltham, MA) using a mixture of Tween 20 and phosphate-buffered saline (0.05% vol/vol). The plates were read at 450 nm using an automated microplate reader (Multiskan; ThermoFisher Scientific). The standard curves were constructed using a 4-parameter logistic regression fitting equation (online software: elisaanalysis.com). The mean intra-assay and inter-assay CV for ICAM-1, PAI-1, and VEGF was <10% and <15%, respectively.

### Statistical analysis

The statistical analyses were performed using the software STATA (version 13; StataCorp, College Station, TX). Based on the stroke and HIV status, 4 clinical profiles were defined: HIV-negative controls, HIV-positive controls, HIV-negative stroke, and HIV-positive stroke. Demographic and clinical parameters were summarized as proportions for categorical variables and median with interquartile range (IQR) for numerical variables. For comparisons across groups, Fisher exact and Kruskall-Wallis tests were used for categorical and numerical variables, respectively.

The third tertile of the distribution of plasma concentration values was considered as the threshold for the distinction between high and low levels for each biomarker. To identify the predictors of endothelial dysfunction, the plasma level of each biomarker was included as a binary dependent variable (low/high levels) in a separate multivariable logistic regression analysis. Age, sex, hypertension, diabetes mellitus, tobacco and alcohol consumption, personal/family history of stroke, ART status, and hypercholesterolemia were included in the model as potential confounders. We also explored the plasma levels of the biomarkers as a continuous variable in a separate multiple linear regression analysis.

To further explore the effect of ART, the regression analyses described above were repeated after excluding HIV-positive patients with a viral load greater than 1,000 RNA copies/mL (25 of 48 HIV-positive participants with viral load available). The cutoff thresholds to distinguish between high and low levels of biomarkers were recalculated to adjust for the change in the population structure and size. The ART status was not included as a confounder in multivariable analyses because 87% of patients with a low or undetectable viral load were on ART.

All the statistical tests performed were 2-tailed, and statistical significance was defined as *p* < 0.05.

### Data availability

The database of the Malawi START study is available for consultation or reuse on request to the principal investigator (L.B.). Sharing of all or part of this database is subject to prior authorization by the University of Malawi College of Medicine Research Ethics Committee.

## Results

### Participants' characteristics

The 229 participants (50.2% women) included in this study were distributed as follows: 136 HIV-negative controls, 32 HIV-positive controls, 42 HIV-negative cases, and 19 HIV-positive cases. The median age of the participants was 55 years (IQR: 38.0–65.0). Participants' baseline characteristics are summarized in table 1.

**Table 1:**
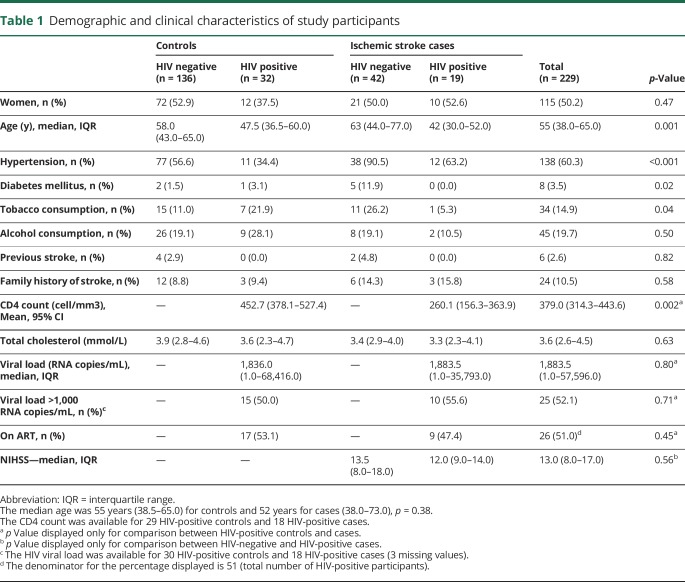
Demographic and clinical characteristics of study participants

The etiologies of 60 of 61 ischemic strokes were as follows: 7 (12%) atherosclerotic strokes, 5 (8%) cardio-thromboembolic strokes, 16 (27%) strokes with other determined causes, and 32 (53%) strokes with an undetermined cause.

### Factors associated with endothelial dysfunction

In the logistic regression analysis, the adjusted OR for having high plasma levels of ICAM-1 was 3.6 (95% CI: 1.3–10.6, *p* = 0.018) for the profile “HIV-positive controls” and 1.2 (95% CI: 0.4–4.0, *p* = 0.772) for the profile “HIV-positive stroke” (table 2). The adjusted OR for having high plasma levels of PAI-1, sTM, or VEGF was not significant for HIV-positive controls and HIV-positive stroke. The adjusted OR for having high plasma levels of PAI-1 was 0.4 (95% CI: 0.1–0.9, *p* = 0.037) for the profile “HIV-negative stroke” (table 2). Linear regression did not modify the associations reported here.

**Table 2:**
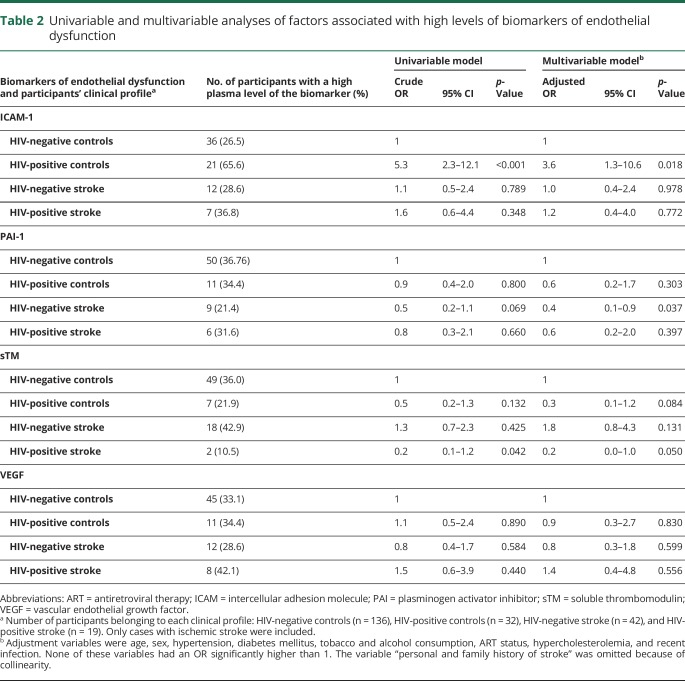
Univariable and multivariable analyses of factors associated with high levels of biomarkers of endothelial dysfunction

After excluding HIV-positive patients with a viral load greater than 1,000 RNA copies/mL, the adjusted OR for having high plasma levels of ICAM-1 was 4.1 (95% CI: 1.3–13.1, *p* = 0.017) for the profile “HIV-positive controls,” whereas the OR for having high plasma levels of PAI-1 was 0.5 (95% CI: 0.2–1.3, *p* = 0.183).

## Discussion

Our results show that HIV infection is independently associated with high plasma levels of ICAM-1, a biomarker of endothelial activation. There was no association between stroke status and high levels of ICAM-1 or between HIV infection and high levels of PAI-1, sTM, and VEGF (table e-1, links.lww.com/NXI/A94).

The association between HIV infection and high levels of ICAM-1 reported here expands the limited literature on sub-Saharan Africa^6^ and is consistent with findings from high-income countries.^2^ Although other risk factors, such as diabetes, hypercholesterolemia, and ART, can contribute to endothelial activation, we found that this association is independent of those factors. Chronic systemic inflammation induced by HIV infection is a trigger for endothelial activation,^7^ which is an important component of endothelial dysfunction. In turn, endothelial activation and dysfunction are both risk factors for atherogenesis. For example, high levels of ICAM-1 have been associated with increasing carotid intima-media thickness, a surrogate marker of atherosclerosis and a predictor of cardiovascular events.^2,8^ In our cohort, more than 50% of the HIV-positive controls were on ART, and their CD4^+^ T-cell count was >400 cells/mm^3^. This means that despite having a relatively stable infection, HIV-infected individuals still have an ongoing endothelial activation as demonstrated by previous studies conducted in Western countries.^9^ Therefore, ART alone might not be enough to suppress cardiovascular risk in people living with HIV, and additional interventions to control HIV-related inflammation might be needed. One potential intervention might be the use of statins as an adjunct therapy to attenuate endothelial dysfunction and vascular inflammation.

Unexpectedly, we showed no association between HIV and endothelial dysfunction among patients with stroke. The most likely explanation is that our sample size was too small, with only 19 HIV-positive stroke cases. Furthermore, despite restricting the analysis to ischemic stroke, the cohort remained heterogeneous, thus reducing our statistical power further and our ability to explore the varied etiologies of cerebrovascular diseases. However, the independent association between HIV infection and high levels of ICAM-1 in the control group suggests that a cardiovascular risk does exist. Consequently, further evaluation in a larger clinical cohort is warranted.

PAI-1 is a molecule that inhibits fibrinolysis. High and low plasma levels are expected in ischemic stroke and hemorrhagic stroke, respectively. A decrease in the plasma concentration of PAI-1 with increasing age has been described.^10^ Therefore, we suspect that the association between levels of PAI-I and the profile “HIV-negative stroke” is due to a residual confounding effect of age.

The absence of a fixed cutoff to distinguish between low and high plasma levels of the biomarkers considered in this study represents a limitation and continues to be a challenge in this field. However, there is good evidence that high plasma concentrations are related to increased cardiovascular risk.^2,11,12^

Overall, this study confirms the association between HIV infection and endothelial activation despite ART in a sub-Saharan African setting. Larger well-phenotyped cohort studies are needed to clarify the contribution of HIV-related endothelial activation to the long-term stroke morbidity and mortality in the ART era.

## Glossary

ART: antiretroviral therapy
CV: coefficient of variability
ICAM: intercellular adhesion molecule
IQR: interquartile range
PAI: plasminogen activator inhibitor
sTM: soluble thrombomodulin
VEGF: vascular endothelial growth factor
